# Claims-based Frailty Index in Japanese Older Adults: A Cohort Study Using LIFE Study Data

**DOI:** 10.2188/jea.JE20220310

**Published:** 2024-03-05

**Authors:** Kiyomasa Nakatsuka, Rei Ono, Shunsuke Murata, Toshihiro Akisue, Haruhisa Fukuda

**Affiliations:** 1 Kobe University Graduate School of Health Sciences, Kobe, Japan; 2 Department of Preventive Medicine and Epidemiology, National Cerebral and Cardiovascular Center Research Institute, Osaka, Japan; 3 Department of Physical Activity Research, National Institutes of Biomedical Innovation, Health and Nutrition, National Institute of Health and Nutrition, Tokyo, Japan; 4 Kyushu University Graduate School of Medical Sciences, Department of Health Care Administration and Management, Fukuoka, Japan

**Keywords:** CFI, claim data, frailty, long-term care insurance, mortality

## Abstract

**Background:**

We aimed to assess whether the United States-developed Claims-based Frailty Index (CFI) can be implemented in Japanese older adults using claims data.

**Methods:**

We used the monthly claims data and certification of long-term care (LTC) insurance data of residents from 12 municipalities from April 2014 to March 2019. The 12 months from first recording was defined as the “baseline period,” and the time thereafter as the “follow-up period”. Participants aged ≥65 years were included, and those with no certified LTC insurance or who died at baseline were excluded. New certification of LTC insurance and all-cause mortality during the follow-up period were defined as outcome events. CFI categorization consisted of three steps including: 1) using 12 months deficit-accumulation approach that assigned different weights to each of the 52 items; 2) the accumulated score to derive the CFI; and 3) categorizing the CFI as “robust” (<0.15), “prefrail” (0.15–0.24), and “frail” (≥0.25). Kaplan–Meier survival curves and Cox proportional hazard models were used to determine the association between CFI and outcomes. Hazard ratios (HRs) and 95% confidence intervals (CIs) were calculated.

**Results:**

There were 519,941 participants in total. After adjusting for covariates, the severe CFI category had a high risk of certification of LTC insurance (prefrail: HR 1.33; 95% CI, 1.27–1.39 and frail: HR 1.60; 95% CI, 1.53–1.68) and all-cause mortality (prefrail: HR 1.44; 95% CI, 1.29–1.60 and frail: HR 1.84; 95% CI, 1.66–2.05).

**Conclusion:**

This study suggests that CFI can be implemented in Japanese claims data to predict the certification of LTC insurance and mortality.

## INTRODUCTION

Frailty is conceptually defined as a clinically recognizable state in which the ability of older people to cope with everyday or acute stressors is compromised by an increased vulnerability brought about by age-associated declines in physiological reserves and function across multiple organ systems.^1^ Some systematic review and meta-analysis studies have showed that 10–24% of community-dwelling older adults were frail, while 13–49% were prefrail,^2^^–^^5^ and the number of frail older adults will increase in the future.^6^ Systematic reviews and population-based studies have showed that frail patients were at high risk of disability, certification of long-term care (LTC) insurance, and all-cause mortality.^4^^,^^7^^,^^8^ The identification of frailty can be used to direct older persons towards appropriate interventions and can assist them in the maintenance of a healthy and independent lifestyle.

Several clinical frailty assessments have been validated. Among them, the frailty phenotype^9^ and deficit accumulation frailty^10^ have been extensively validated and widely used. The frailty phenotype generally offers better clinical operationalization, based on weight loss, exhaustion, inactivity, slowness, and weakness. Deficit accumulation frailty is defined by the proportion of deficits from a list of age-associated health deficits. Both phenotype and deficit accumulation frailty could assess the risk of LTC insurance certification and all-cause mortality.^11^^,^^12^ The Claims-based Frailty Index (CFI) quantifies frailty using claims data with a deficit accumulation approach.^13^ Previous studies have validated CFI as another frailty scale.^14^^,^^15^ Generally, frailty assessments require measurements that are not performed during daily visits to clinics or hospitals. CFI can be measured using data from routine visits without participating in frail measurements or answering questionnaires. Thus, CFI have been suggested to identify frailty without individual efforts.^14^^,^^15^ For policymakers in municipalities, CFI can determine the frailty of many residents and might contribute to municipal health policy development. Moreover, as the CFI can be calculated using only claims data, the measurement biases in the CFI would be smaller than those in the other frailty scales.

Japan has a universal insurance system, and the selection bias in Japanese claims data studies may be lower than that in other registry data studies or claims data studies from other countries. Implementing CFI in Japanese claims data could provide information reflecting real-world practice with a higher degree of accuracy than previous studies. However, it is unclear whether CFI can be implemented using Japanese claims data.

This study aimed to investigate whether CFI could be implemented in Japanese older adults to predict the certification of LTC insurance and all-cause mortality.

## METHODS

This retrospective study was conducted using a database produced by the Longevity Improvement & Fair Evidence (LIFE) Study.^16^ The LIFE study is a longitudinal cohort study that collects administrative claims data from residents in participating municipalities. The database included information from National Health Insurance enrollees (comprising mainly retirees aged ≤74 years, self-employed people, non-regularly employed people, and primary industry workers), Latter-Stage Elderly Healthcare system enrollees (comprising all residents aged ≥75 years), and LTC insurance enrollees (comprising residents aged ≥65 years with certified care needs and residents aged 40–64 years with a debilitating disease, such as dementia) from April 2014 onward. The National Health Insurance and Latter-Stage Elderly Healthcare systems cover medical care services, while the LTC insurance system covers LTC services. The monthly participation by the municipalities in the LIFE study varied. The LIFE Study database contains administrative claims data from various municipalities, including the urban and rural areas, on the vast majority of older adults living in each municipality.^16^

This study was approved by the Kyushu University Institutional Review Board for Clinical Research (approval number: 2021-399).

### Study subjects

**Figure 1.**
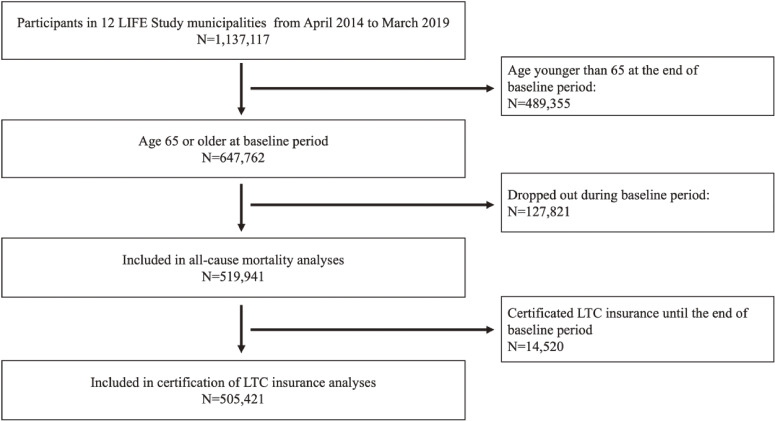
Flowchart of the participants

Claims data from April 2014 to March 2019 were used in this study. Participants were selected from the National Health Insurance enrollees and the Latter-stage Elderly Healthcare system enrollees residing in 12 LIFE study municipalities. We defined the 12 months from the first recording as the “baseline period,” and the time thereafter as the “follow-up period,” in accordance with previous studies using CFI.^15^^,^^17^^,^^18^ The 12-month baseline period data were used to assess analyze association between CFI and other charactetristics.^15^^,^^17^^,^^18^ A total of 1,137,117 participants resided in the 12 LIFE study municipalities. Of these, 647,762 met the inclusion criteria (age of ≥65 years at the end of the baseline period). Then, 14,520 participants dropped out during the baseline period (reasons for dropout included: moved to other cities, resignation of their insurance, or death). The final analyses were conducted on 519,941 participants. The flowchart is shown in Figure 1.

### Outcomes

The outcome events were new certification of LTC insurance and all-cause mortality during the follow-up period. LTC insurance was introduced as part of Japan’s social security policy reform to address the prolonged economic slump and soaring medical and LTC expenditures for older people. The LTC includes seven levels: support levels 1 and 2 and care need levels 1 to 5.^19^^,^^20^ Participants who had already been certified for LTC insurance in the baseline period were excluded from the LTC analyses.

### Claims-based Frailty Index (CFI)

CFI was developed using the International Classification of Diseases, 10th version (ICD-10). CFI calculation was conducted using the baseline period claims data. The baseline period data were checked for the occurrence of each of the 52 items, which were weighted according to previous studies.^13^ The accumulated score was defined as the CFI. The original CFI code was based on the ICD-10 codes as well as the United States treatment codes. These treatment codes differ from Japanese codes. Thus, by referring to previous studies,^13^ we adjusted the weights of each variable, which are shown in eTable 1. The ICD-10 codes for the CFI are available at https://github.com/KiyomasaNakatsuka/CFIcode.git. CFI can range from 0 to 1, with higher values indicating greater frailty. Based on previous studies,^14^^,^^15^ we categorized frailty status as “robust” (<0.15), “prefrail” (0.15–0.24), and “frail” (≥0.25). In the CFI calculations, ICD-10 codes with suspected diagnoses were ignored. In Japan, a suspected diagnosis is attached when doctors consider a diagnosis to be temporary or likely. Doctors often make suspected diagnoses to levy medical fees for performing blood tests and prescriptions.

### Other variables

Data on age, sex, prevalence of comorbidities, history of admission, income, and the total cost of medical care were extracted from the claims data in the baseline period. Comorbidities included cancer, chronic obstructive pulmonary disease (COPD), diabetes mellitus (DM), stroke, and dementia. Comorbidities were prevalent when ICD-10 codes without a suspected diagnosis were recorded at least once in the baseline period. The same method was used for the history of admission and comorbidities. The total cost of medical care was defined using the all-claims data in baseline period. The sum of the cost was analyzed as a numerical variable. The income variable was determined based on the copayment limit by the burden of medical cost per month, which depends on the household income and age. In the LIFE Study database, the information of the copayment limit by the burden of medical cost per month included the detail classification of low income, and this information was connected in most receipts. The participants whose annual household income was >800,000 yen but <1,600,000 yen were classified as the “low-income” group, while those with an income ≤800,000 yen were classified as the “very-low-income” group.

### Statistical analysis

The prevalence of the CFI category and outcomes was described as percentages and 95% confidence intervals (CIs). Categorical variables (sex, prevalence of comorbidities, income, and outcome) were described as numbers and proportions. Continuous variables are described as means and standard deviations (SDs). All variables were stratified by CFI category (robust, prefrail, and frail).

We estimated the outcome incidences for the CFI category using Kaplan–Meier survival curves and compared them using the log-rank test. Cox proportional hazards models were used to determine the effects of the CFI category on the outcomes. The outcomes were the certification of LTC insurance and all-cause mortality. Exposure was the CFI category. We censored participants when their insurance data terminated (moved or resignation of their insurance) or on March 31, 2019, whichever came first. Three models were conducted: 1) univariable model, 2) model adjusted for covariates (age and sex); and 3) model adjusted for the history of admission, income, and total cost of medical care, in addition to the variables added as covariates to model 2.

### Sensitivity analysis

In addition to the main analysis, we conducted four sensitivity analyses. First, the quantile of CFI score was used as the exposure variable to assess the robustness of CFI classification into “robust,” “prefrail,” and “frail.” Second, LTC insurance was defined as LTC Care Need level 2 or above. LTC Care Need Level 2 is defined as the requirement for assistance in at least one basic activity of daily living (ADL) task.^21^ Third, as the three municipalities claims data included the medical claims data only (the three municipalities did not have LTC-related data), the analysis for all-cause mortality was performed using data from 15 LIFE Study municipalities. Lastly, the analyses based on the data from each of the 12 municipalities were conducted to determine the difference between CFI categories in each municipality.

Hazard ratios (HRs) and 95% CIs were calculated for all analyses. Statistical significance was defined as two-sided *P* < 0.05. All statistical analyses were performed using R version 4.1.3 (R Foundation for Statistical Computing, Vienna, Austria) and “survival” packages.^22^^,^^23^

## RESULTS

Table 1 shows the baseline characteristics, comorbidities, and incidence of outcomes according to the frailty status. Overall, there were 519,941 participants with 270,438 (52.0%), 161,128 (31.0%), and 88,375 (17.0%) in the robust (CFI <0.15), prefrail (CFI 0.15–0.24) and frail (CFI ≥0.25) categories, respectively. The mean age in robust, prefrail, and frail groups were 72.9 (SD, 7.1), 77.0 (SD, 7.9), and 82.5 (SD 7.9) years, respectively.

**Table 1.** The characteristics of participants according to the CFI category (*n* = 519,941)

	Robust *n* = 270,438 (52.0%)		Prefrail *n* = 161,128 (31.0%)		Frail *n* = 88,375 (17.0%)	
Age, years, mean [SD]	72.88	[7.06]	77.03	[7.88]	82.51	[7.90]
Female, *n* (%)	156,234	(57.8)	97,101	(60.3)	49,823	(56.4)
Income						
Low income, *n* (%)	3,230	(1.2)	4,639	(2.9)	8,439	(9.5)
Very low income, *n* (%)	112	(0.04)	270	(0.2)	560	(0.6)
Admission, *n* (%)	320	(0.1)	1,511	(0.9)	2,785	(3.2)
Cancer, *n* (%)	3,689	(1.4)	8,970	(5.7)	6,971	(7.9)
COPD, *n* (%)	498	(0.2)	1,430	(0.9)	2,448	(2.8)
DM, *n* (%)	3,909	(1.4)	6,871	(4.3)	8,874	(10)
Dementia, *n* (%)	2,034	(0.8)	3,151	(2.0)	3,960	(4.5)
Cost of medical care, Japanese yen, mean [SD]	13,890	[2,409]	17,893	[3,699]	19,808	[4,001]
Mortality, *n* (%)	632	(0.2)	596	(0.4)	608	(0.7)
Certificated LTC insurance, *n* (%)	4,499	(1.4)	3,966	(2.2)	4,095	(4.6)

CFI, Claims-based Frailty Index; COPD, chronic obstructive pulmonary disease; DM, diabetes mellitus; LTC, long-term care; SD, standardized deviation.

Kaplan–Meier curves of the CFI category and cumulative incidence of LTC insurance certification are shown in Figure 2A. The rate of LTC insurance certification increased in the severe CFI category. The number of LTC insurance certifications in the robust, prefrail, and frail groups was 4,499, 3,966, and 4,095, respectively. The 4-year cumulative incidence in the robust, prefrail, and frail groups was 4.7% (95% CI, 3.7–5.6%), 9.2% (95% CI, 8.1–10.2%), and 12% (9.2–15.4%), respectively. Table 2 shows the results of LTC insurance certification. The severe CFI category had a higher risk of LTC insurance certification than the robust group (prefrail: HR 1.30; 95% CI, 1.15–1.45 and frail: HR 1.78; 95% CI, 1.67–1.90). After adjusting for covariates, the trends were not changed in models 2 (prefrail: HR 1.26; 95% CI, 1.08–1.43 and frail: HR 1.71; 95% CI, 1.51–1.91) and 3 (prefrail: HR 1.30; 95% CI, 1.14–1.46 and frail: HR 1.58; 95% CI, 1.30–1.86).

**Figure 2A.**
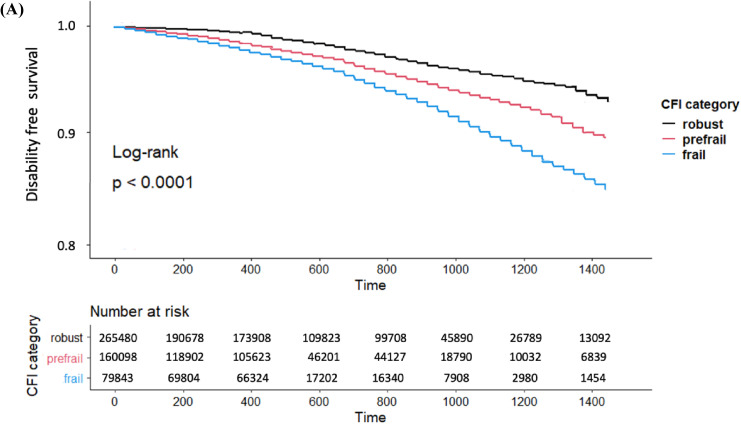
The Kaplan–Meier curves of CFI category and cumulative incidence of LTC insurance certification

**Table 2.** The effect of CFI category at baseline period on the outcomes

	Certification of LTC insurance						All-cause mortality					

	Model 1^a^		Model 2^b^		Model 3^c^		Model 1^a^		Model 2^b^		Model 3^c^	
	
	HR	95% CI	HR	95% CI	HR	95% CI	HR	95% CI	HR	95% CI	HR	95% CI
Robust	1	—	1	—	1	—	1	—	1	—	1	—
Prefrail	1.30	1.15–1.45	1.26	1.08–1.43	1.30	1.14–1.46	1.90	1.65–2.15	1.39	1.20–1.59	1.43	1.25–1.62
Frail	1.78	1.67–1.90	1.71	1.51–1.91	1.58	1.30–1.86	2.70	2.30–3.09	1.90	1.62–2.17	1.84	1.63–2.05

CI, confidence interval; CFI, Claims-based Frailty Index; HR, hazard ratio; LTC, long-term care.

^a^Model 1: univariable model.

^b^Model 2: adjusted the covariates, age, and gender.

^c^Model 3: admission, income, and total cost of medical care in baseline period were added to model 2.

The Kaplan–Meier survival curves of the CFI category and all-cause mortality are shown in Figure 2B. The survival rate was decreased in the severe CFI category. Table 2 shows the results for all-cause mortality. The number of all-cause mortality cases in the robust, prefrail, and frail groups was 632, 596, and 608, respectively. The 4-year survival rates in the robust, prefrail, and frail groups were 99.3% (95% CI, 98.9–99.8%), 98.5% (97.9–99.1%), and 97.8% (97.0–98.6%), respectively. The severe CFI category had a higher risk of all-cause mortality than the robust group (prefrail: HR 1.90; 95% CI, 1.65–2.15 and frail: HR 2.70; 95% CI, 2.30–3.09) in model 1. After adjusting for covariates, the trends did not change in model 2 (prefrail: HR 1.39; 95% CI, 1.20–1.59 and frail: HR 1.90; 95% CI, 1.62–2.17) and model 3 (prefrail: HR 1.43; 95% CI, 1.25–1.62 and frail: HR 1.84; 95% CI, 1.63–2.05).

**Figure 2B.**
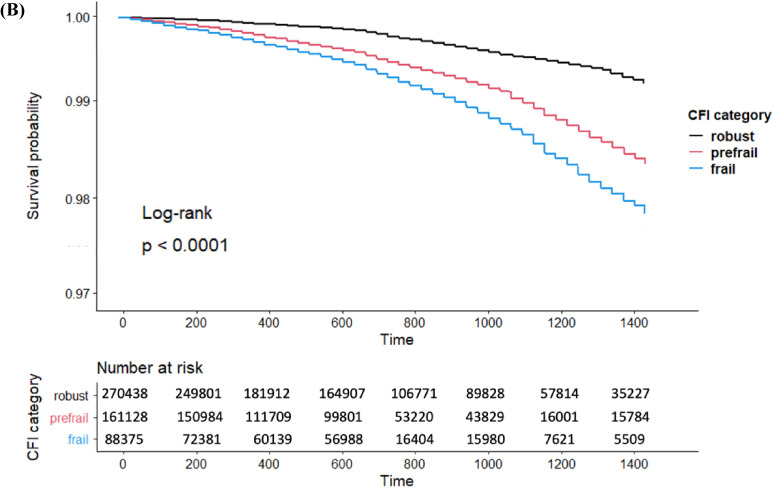
The Kaplan–Meier survival curves of CFI category and all-cause mortality

The results of the sensitivity analyses are presented in eTable 2, eTable 3, eTable 4, eTable 5, and eTable 6. The results of the analyses using the quantile of CFI as exposure are presented in eTable 1 and eTable 2. As in the main analyses, the severe CFI category had a high risk of outcome incidence. The results of the analysis using LTC Care Need Level 2 or above as outcomes are shown in eTable 3. The same trends were obtained in the analysis using LTC Care Need Level 2 or above. The results of the analysis performed based on the data from the 15 municipalities are presented in eTable 5. The same trends were shown. eTable 6A and eTable 6B show the results of the analyses conducted using the data from each municipality. Most results showed similar trends compared to those of the main analysis.

## DISCUSSION

This study investigated whether the CFI category was associated with LTC insurance certification and all-cause mortality using the LIFE study database. Among Japanese older adults, the CFI was categorized as robust in approximately 54%, prefrail in 31%, and frail in 15%. The severe CFI category had a high risk for LTC insurance certification and all-cause mortality.

Previous systematic reviews and meta-analyses have shown the prevalence of prefrail and frail populations as assessed by the deficit accumulation frailty index^24^^,^^25^^,^^32^ to be 28–54% and 14–26%, respectively. These prevalence rates are similar to the deviations observed in this study. Moreover, in several previous studies that assessed frailty using claims data,^15^^,^^26^^–^^28^ the prefrail and frail populations were 8.9–39% and 16–54%, respectively. These prevalence rates are also similar to ours.

Previous studies have indicated that severe CFI is associated with a high risk of ADL disability.^14^^,^^15^ These results are consistent with those of this study. In previous Japanese studies, frailty assessed using physical measurements or questionnaires was found to be a risk factor for LTC insurance.^7^^,^^11^^,^^29^ The results of these Japanese studies have shown the HRs of the certification of LTC insurance were 1.28 to 2.35 and 1.25 to 5.07 in the prefrail and frail populations, respectively, compared to the robust population. These HRs were similar to the results of this study (HR in prefrail was 1.29, and HR in frail was 1.58). Thus, this study suggests that the CFI can predict the certification of LTC insurance in Japanese older adults, as with other frailty scales.

Severe CFI has been shown to be a high risk for all-cause mortality,^6^^,^^13^ and these results are similar to those of the current study. In previous Japanese studies, frailty was found to be a risk factor for all-cause mortality.^7^^,^^30^^,^^31^ The results of these Japanese studies showed that the HRs of all-cause mortality were 1.44 to 2.34 and 1.58 to 5.40 in the prefrail and frail populations, respectively, compared to those in the robust population. These HRs were also similar to the results of this study (HR in pre-frail was 1.43, and HR in frail was 1.81). Thus, this study suggests that CFI can also predict all-cause mortality in the Japanese older adults, as with other frailty scales.

The severe CFI category had a high risk for LTC insurance certification and all-cause mortality. CFI assesses the risk of LTC insurance certification and all-cause mortality without individual efforts. In general, frailty assessments require special questionnaires or measurements that are not conducted during routine visits to clinics or hospitals. CFI can be calculated using the data of daily visits to clinics or hospitals without special assessments of frailty. Therefore, the CFI might be an indicator of prioritizing intervention conducted by public health services and an indicator of policymaking to prevent frailty. Moreover, frailty assessments using CFI could decrease the recall bias. Generally, frailty assessments often require individual information or recent body changes, including instrumental ADL and weight change over the last 6 months. These items may have caused recall bias. As the CFI can be calculated without recall, accurate frailty can be measured.

### Limitations

This study had three limitations. First, individuals who did not receive any medical services were not included in this study. Furthermore, older adults who were healthcare non-users could not be considered in this study. However, because Japan has a national health insurance (NHI) system, over 75% older adults have health insurance for medical services and can afford frequent visits at low medical costs. Most older adults in the LIFE study municipalities receive medical services. Thus, selection bias was small compared to those in studies recruiting participants. In the future, studies should be conducted using population registry data to decrease selection bias. Second, CFI calculation was not performed perfectly as the original calculations. The treatment codes in the United States were different from Japanese codes. Thus, some treatment codes were not included in the CFI calculation and there might be misclassification in CFI categorization. However, the weights of other codes were modified to equalize the total CFI score in this study and the original CFI score. The deviations of frailty and prefrailty in previous systematic reviews and meta-analyses were similar to those in this study.^3^^,^^24^^,^^25^ Moreover, some previous Japanese studies that investigated frailty using validated scales reported robust, prefrail, and frail populations of 26–56%, 28–65%, and 4.6–39%, respectively.^30^^–^^32^ These deviations of robust, prefrail, and frail categories are similar to those of the CFI category in this study (robust, prefrail, and frail were approximately 54%, 31%, and 15%, respectively). The CFI category in this study should be compared with validated frailty scale in future study. Third, death was obtained from claims data, not from official death information, such as death registration. A previous Japanese study showed that the validity of death information in inpatient claims was high, suggesting its potential usefulness in identifying death.^33^ Thus, most of the people who died in the hospital were recorded. Further studies should use both claims and official death data.

### Conclusion

This retrospective study suggests that severe CFI is a risk factor for LTC insurance certification and all-cause mortality in Japanese older adults. The CFI can be implemented in Japanese claims data, as with claims data in other countries.

## SUPPLEMENTARY MATERIAL

eTable 1. each weight of CFI codes

eTable 2. The effect of CFI quantile at baseline period on LTC insurance certification

eTable 3. The effect of CFI quantile at baseline period on all-cause mortality

eTable 4. The effect of CFI category at baseline period on the certification of LTC insurance Care Need level 2 and above

eTable 5. The effect of CFI category at baseline period on all-cause mortality in 15 municipalities

eTable 6A. The effect of CFI category at baseline period on the certification of LTC insurance in each municipality (reference: robust)

eTable 6B. The effect of CFI category at baseline period on all-cause mortality in each municipality (reference: robust)
