# Supplementary material for: Claims-based Frailty Index in Japanese Older Adults: A Cohort Study Using LIFE Study Data
Source: J Epidemiol. 2024 Mar 5;34(3):112–8. doi: 10.2188/jea.JE20220310 (PMC10853043; doi:10.2188/jea.JE20220310)
Supplement: Supplementary file 1 [file je-34-112-s001.pdf]

eTable 1. each weight of CFI codes

CFI code	weight
1	0.0102491879504267
2	0.0209210228266441
3	0.0146341498055061
4	0.0140353999939692
5	-0.00903407803877816
6	-0.00107422760305159
7	0.00773091668425655
8	0.0189838910834364
9	-0.00993220275608359
10	0.0149687452884238
11	-0.0141586720139915
12	-0.0119749962307391
13	0.0835608192865543
14	0.0371929294695896
15	0.0238267204414558
16	0.0709870732442783
17	0.00722021831559268
18	0.00595227753822031
19	-0.00107422760305159
20	0.0306419021198323
21	0.0338117540632633
22	0.0350268639749118
23	0.0273311678678045
24	0.00186669058890933
25	0.0217310961010765
26	-0.00915735005880047
27	0.0208681919609203
28	0.0228053237041281
29	0.00459628531797485
30	0.000880514428730815
31	0.00683279196695112
32	0.0184379721376233
33	0.0187021264662425
34	-0.0375275249525073
35	0.00987937189035974
36	-0.000933345294454663
37	0.0247952863130597
38	0.0173285239574224
39	0.000369816060066942
40	0.00685040225552574
41	-0.0147222012483792
42	0.00128555106594699
43	0.0104429011247475
44	-0.00406797666073636
45	0.0197059129149956
46	0.0359602092693665
47	0.0189134499291379
48	-0.00250066097759551
49	-0.00332834454060248
50	0.00336356511775171
51	0.00207801405180472
52	-0.0212732285981365

CFI, Claims-based Frailty Index.

eTable 2. The effect of CFI quantile at baseline period on LTC insurance certification

			Model 1[a]		Model 2[b]		Model 3[c]	
	number of participants	certification of LTC insurance	HR	95% CI	HR	95% CI	HR	95% CI
<25th percentile CFI <0.11	124,670	1,598	1		1		1	
<50th percentile 0.11≤ CFI <0.18	125,409	2,754	1.54	1.46–1.63	1.30	1.19–1.37	1.29	1.20–1.39
<75th percentile 0.18≤ CFI <0.27	125,070	3,812	2.03	1.93–2.13	1.52	1.42–1.63	1.50	1.39–1.62
75th percentile ≤ 0.27≤ CFI	130,272	4,396	2.49	2.36–2.63	1.78	1.60–1.96	1.72	1.61–1.83

CI, confidence interval; CFI, Claims-based Frailty Index; HR, hazard ratio; LTC, long-term care.

[a]Model 1: univariable model.

[b]Model 2: adjusted the covariates, age, and gender.

[c]Model 3: admission, income, and total cost of medical care in baseline period were added to model 2.

eTable 3. The effect of CFI quantile at baseline period on all-cause mortality

	number of participants	all-cause mortality	Model 1[a]		Model 2[b]		Model 3[c]	
			HR	95% CI	HR	95% CI	HR	95% CI
<25th percentile CFI <0.11	132,870	255	1	-	1	-	1	-
<50th percentile 0.11≤ CFI <0.18	129,738	475	1.96	1.70–2.22	1.54	1.29–1.74	1.52	1.28–1.74
<75th percentile 0.18≤ CFI <0.27	126,089	518	2.71	2.42–3.01	1.90	1.67–2.20	1.88	1.66–2.24
75th percentile ≤ 0.27≤ CFI	131,244	588	3.69	3.18–4.30	2.89	2.34–3.50	2.81	2.42–3.33

CI, confidence interval; CFI, Claims-based Frailty Index; HR, hazard ratio; LTC, long-term care.

[a]Model 1: univariable model.

[b]Model 2: adjusted the covariates, age, and gender.

[c]Model 3: admission, income, and total cost of medical care in baseline period were added to model 2.

eTable 4. The effect of CFI category at baseline period on the certification of LTC insurance Care Need level 2 and above

	Model 1[a]		Model 2[b]		Model 3[c]	
	HR	95% CI	HR	95% CI	HR	95% CI
Robust	1		1		1	
Prefrail	1.80	1.65–1.94	1.43	1.36–1.50	1.45	1.38–1.52
Frail	2.59	2.48–2.71	1.81	1.73–1.89	1.78	1.70–1.86

CI, confidence interval; CFI, Claims-based Frailty Index; HR, hazard ratio; LTC, long-term care.

[a]Model 1: univariable model.

[b]Model 2: adjusted the covariates, age, and gender.

[c]Model 3: admission, income, and total cost of medical care in baseline period were added to model 2.

eTable 5. The effect of CFI category at baseline period on all-cause mortality in 15 municipalities

	All-cause mortality					
	Model 1[a]		Model 2[b]		Model 3[c]	
	HR	95% CI	HR	95% CI	HR	95% CI
Robust, n=318,245	1	-	1	-	1	-
Prefrail, n=182,513	1.82	1.62–2.01	1.43	1.30–1.58	1.48	1.28–1.69
Frail, n=89,516	2.64	2.30–2.98	1.87	1.65–2.08	1.81	1.63–2.02

CI, confidence interval; CFI, Claims-based Frailty Index; HR, hazard ratio; LTC, long-term care.

[a]Model 1: univariable model.

[b]Model 2: adjusted the covariates, age, and gender.

[c]Model 3: admission, income, and total cost of medical care in baseline period were added to model 2.

eTable 6A. The effect of CFI category at baseline period on the certification of LTC insurance in each municipality (reference: robust)

	City A	City B	City C	City D	City E	City F	City G	City H	City I	City J	City K	City L
	HR<br>95% CI	HR<br>95% CI	HR<br>95% CI	HR<br>95% CI	HR<br>95% CI	HR<br>95% CI	HR<br>95% CI	HR<br>95% CI	HR<br>95% CI	HR<br>95% CI	HR<br>95% CI	HR<br>95% CI
Prefrail: Model 3[a]	1.46<br>1.20–1.80	1.29<br>1.14–1.44	1.43<br>1.14–1.85	1.40<br>1.14–1.70	1.01<br>0.76–1.27	1.10<br>0.89–1.24	1.10<br>0.90–1.30	1.15<br>1.10–1.20	1.45<br>1.26–1.65	1.37<br>1.21–1.63	1.39<br>1.19–1.61	1.10<br>0.89–1.33
Frail: Model 3[a]	1.97<br>1.48–2.52	1.58<br>1.30–1.86	2.17<br>1.65–2.63	1.88<br>1.30–2.51	1.18<br>1.00–1.40	2.03<br>1.65–2.60	1.45<br>1.22–1.72	1.58<br>1.30–1.87	1.85<br>1.50–2.26	1.88<br>1.60–2.16	1.80<br>1.50–2.15	1.42<br>1.10–1.79

CI, confidence interval; CFI, Claims-based Frailty Index; HR, hazard ratio; LTC, long-term care.

[a]Model 3: admission, income, and total cost of medical care in baseline period were added to model 2.

eTable 6B. The effect of CFI category at baseline period on all-cause mortality in each municipality (reference: robust)

	City A	City B	City C	City D	City E	City F	City G	City H	City I	City J	City K	City L
	HR<br>95% CI	HR<br>95% CI	HR<br>95% CI	HR<br>95% CI	HR<br>95% CI	HR<br>95% CI	HR<br>95% CI	HR<br>95% CI	HR<br>95% CI	HR<br>95% CI	HR<br>95% CI	HR<br>95% CI
Prefrail: Model 3[a]	1.18<br>1.00–1.40	1.43<br>1.28–1.59	1.79<br>0.89–2.69	1.11<br>0.79–1.59	1.00<br>0.74–1.28	1.43<br>1.11–1.78	1.57<br>1.20–2.05	1.43<br>1.28–1.59	1.56<br>1.34–1.86	1.55<br>1.28–1.89	1.23<br>0.98–1.55	1.36<br>1.14–1.64
Frail: Model 3[a]	2.18<br>1.81–2.58	1.81<br>1.63–2.02	1.90<br>1.04–2.76	1.31<br>0.89–1.75	1.57<br>0.90–2.32	1.71<br>1.47–2.00	1.81<br>1.40–2.30	1.81<br>1.63–2.02	2.23<br>1.80–2.74	1.81<br>1.60–2.08	2.30<br>1.99–2.79	1.81<br>1.41–2.29

CI, confidence interval; CFI, Claims-based Frailty Index; HR, hazard ratio.

[a]Model 3: admission, income, and total cost of medical care in baseline period were added to model 2.
